# Oxidative stress and mitochondrial damage: importance in non-SOD1 ALS

**DOI:** 10.3389/fncel.2015.00041

**Published:** 2015-02-17

**Authors:** Maria Teresa Carrì, Cristiana Valle, Francesca Bozzo, Mauro Cozzolino

**Affiliations:** ^1^Department of Biology, Università di Roma Tor VergataRome, Italy; ^2^Fondazione Santa Lucia, IRCCSRome, Italy; ^3^Institute of Cell Biology and Neurobiology, IBCN, National Research Council, CNRRome, Italy; ^4^Institute of Translational Pharmacology, National Research Council, CNR, Molecular Mechanisms of Neurodegenerative DiseasesRome, Italy

**Keywords:** Amyotrophic Lateral Sclerosis, ALS, mitochondria, oxidative stress, neurodegeneration

## Abstract

It is well known that mitochondrial damage (MD) is both the major contributor to oxidative stress (OS) (the condition arising from unbalance between production and removal of reactive oxygen species) and one of the major consequences of OS, because of the high dependance of mitochondrial function on redox-sensitive targets such as intact membranes. Conditions in which neuronal cells are not able to cope with MD and OS seem to lead or contribute to several neurodegenerative diseases including Amyotrophic Lateral Sclerosis (ALS), at least in the most studied superoxide dismutase 1 (SOD1)-linked genetic variant. As summarized in this review, new evidence indicates that MD and OS play a role also in non-SOD1 ALS and thus they may represent a target for therapy despite previous failures in clinical trials.

## Introduction

Amyotrophic Lateral Sclerosis (ALS) is a fatal motor neuron disease occurring less than once in 10000 people and thus classified as a rare disease. ALS is not purely a motor neuron disease and neuroinflammation and muscle degeneration also contribute to the non-cell autonomous death of motor neurons. As for other neurodegenerative diseases, ALS is sporadic in most cases, with a clear genetic origin in about 10% of patients, and is clinically heterogeneous in age and site of disease onset as well as rate of disease progression.

Different mechanisms such as protein aggregation, oxidative stress (OS), mitochondrial damage (MD), excitotoxicity and RNA dysmetabolism seem to contribute to ALS. Evidence on the relevance of these mechanisms comes in large part from models based on the expression of ALS-linked mutant superoxide dismutase 1 (SOD1). However, mutant SOD1 causes less than 1% of ALS cases and the list of mutant proteins found in ALS patients has grown noticeably in the last decade (Marangi and Traynor, 2014), to include a number of proteins related to cell functions as diverse as RNA metabolism (TDP-43, FUS/TLS, Senataxin, Ataxin2, HNRNPA2/B1, ELP3, HNRNPA1), vesicle trafficking (Alsin, FIG4, OPTN, VABP, CHMP2B) and proteasomal function (UBQLN2, VCP). Noticeably, almost half of the familial cases are linked to a non-coding sequence, i.e., to an expanded hexanucleotide repeat in the C9orf72 gene.

Because mutations in SOD1, an ubiquitous superoxide scavenging enzyme, were the first identified cause of familial ALS in 1993, OS has been an obvious candidate to explain the pathogenesis of this disease. MD was also demonstrated in patients and in mutant SOD1 models soon after (reviewed in Cozzolino et al., 2012) and the link between the two, OS and MD, appeared most probable, if not obvious.

We will now briefly review recent evidence that OS and MD are found also in non-SOD1 linked ALS.

## Oxidative stress in ALS

OS biomarkers have been repeatedly found in sporadic ALS patients, which may indicate that abnormal OS is relevant to the pathogenesis of this disease (D’Amico et al., 2013). A number of environmental factors may contribute to the generation of noxious free radicals, both Reactive Oxygen Species (ROS) and Reactive Nitrogen Species (RNS). For instance, transition metal-mediated OS has been proposed to contribute to ALS pathogenesis more than 10 years ago (reviewed in Carrí et al., 2003) and the role of metals is still discussed (Lovejoy and Guillemin, 2014). While no single pro-oxidant factor has emerged as crucially linked to sporadic ALS, recent genetic evidence reinforces the concept that OS may play a main role in familial ALS including in non-SOD1 ALS. For instance, expression of mutant valosin-containing protein (VCP, a member of the type II AAA+ ATPase family with a number of cellular functions including mitochondrial quality control, autophagy, vesicle transport and fusion, 26 S proteasome function, and assembly of peroxisomes) found in ALS patients makes human neuroblastoma SH-SY5Y cells susceptible to OS induced by treatment with L-buthionine sulfoximine, an inhibitor of the synthesis of glutathione (GSH; Hirano et al., 2014). GSH is a free-radical scavenger tripeptide which is among the main regulators of the intracellular redox state. Its levels are lower in the motor cortex of ALS patients than in healthy volunteers *in vivo* (Weiduschat et al., 2014) and decreasing GSH accelerates neurological deficit and mitochondrial pathology in the mutant SOD1 ALS mice model (Vargas et al., 2011). Furthermore, GSH depletion in cultured neurons induces the formation of cytoplasmic TDP-43 inclusions which are found in sporadic ALS patients (Iguchi et al., 2012).

Alterations in markers of OS other than GSH are found as a consequence of the expression of ALS-linked mutations. For instance, expression of mutant TDP-43 in a motor neuron-like cell line induces OS, MD and nuclear accumulation of nuclear factor E2-related factor 2 (Nrf2) that is an indicator and modulator of OS (Duan et al., 2010); in a yeast model, TDP-43-expressing cells display increased markers of OS, apoptosis, and necrosis and these cytotoxic effects are potentiated upon expression of disease-associated variants (Braun et al., 2011) and *Drosophila* flies engineered for motoneuron-directed expression of TDP-43 have increased levels of protein carbonylation and Glutathione S transferase D1, both known markers of OS (Zhan et al., 2015). Overall these data indicate that OS is important also for TDP-43-triggered cell death, although the mechanism is still debated.

OS may not only increase the net production of ROS and RNS, but also affect protein conformation and structure, leading to the accumulation of the abnormal protein inclusions that are extensively described in ALS mouse models and patient-derived tissue. Cysteine-mediated aggregation of mutant SOD1 has been widely studied and wild type SOD1 also hyper-aggregates when oxidized (Guareschi et al., 2012). Treatments that deplete the cellular pool of GSH exacerbate mutSOD1s insolubility, whereas an overload of intracellular GSH or overexpression of glutaredoxins 1 and 2 significantly rescues mutSOD1s solubility in the cytosol and in mitochondria, respectively (Ferri et al., 2010). Interestingly, recent evidence suggests that also wild-type and mutant TDP-43 aggregation is caused by incorrect disulphide bonds involving Cys residues in one of its RNA recognition motifs, and that aggregation is promoted by OS (Cohen et al., 2012; Shodai et al., 2013).

Aggregation of TDP-43 (Parker et al., 2012) and FUS (Gerbino et al., 2013; Vance et al., 2013) proceeds, at least in part, through the stress granules (SGs) pathway. SGs are highly dynamic structures that are formed upon OS and contain RNA-binding proteins, transcription factors, RNA helicases and nucleases that work as sorting granules for mRNAs undergoing degradation, storage or translation (Baron et al., 2013 and references therein). SGs are found as a consequence of FUS mutation and mutated FUS is more rapidly directed to SGs after OS than wild type FUS (Bosco et al., 2010). Furthermore, TDP-43 is recruited to SGs in conditions of OS (Colombrita et al., 2009). These SGs may simply sequester a subset of mRNAs thus inducing cell dysfunction or serve as nucleation site for larger protein aggregates as the ones found in ALS patients.

Possibly representing a cellular response to this kind of aggregation and as a reaction against ER stress and unfolded protein response (UPR), protein disulfide isomerase (PDI) expression levels are upregulated in spinal cord tissue of ALS patients where PDI co-localizes with SOD1, TDP-43, and FUS (Atkin et al., 2008; Honjo et al., 2011; Farg et al., 2012; Walker et al., 2013). PDI co-localizes also with VAPB inclusions in a *Drosophila melanogaster* model of ALS (Tsuda et al., 2008). However, PDI itself contains an active site thiol group that is necessary for its activity and it is found oxidized through S-nitrosylation in ALS (Walker et al., 2010), which may lead to a cycle of PDI upregulation and inactivation that perpetuates redox dysregulation and protein aggregation.

Interestingly, even mutant C9orf72 repeats may be related to OS. Indeed, similarly to what happens when expressing mutant SOD1, motor neurons differentiated from patients’ iPSC (induced Pluripotent Stem Cells) and expressing expanded C9orf72 repeat display a significant induction of catalase, which is indicative of OS, and a significant change in levels of the mitochondrial transporter MTX3 (Kiskinis et al., 2014).

Mitochondria are a known target of OS because of their high dependance on membrane integrity and because they possess their own DNA and RNA that may be damaged by oxidation as well.

## Mitochondrial damage in ALS

Keeping in mind that mitochondria are not only a target of OS, but also the main site of production of ROS, it is obvious that OS may result from impairment of mitochondrial function.

A number of studies have reported altered mitochondrial respiratory complex activity in ALS tissues including postmortem brain and spinal cord tissue, patient lymphocytes, and in the SOD1 transgenic mouse model of ALS (for a review Cozzolino and Carrì, 2012; Tan et al., 2014). MD may follow the accumulation of aggregated SOD1 associated to mitochondria (Ferri et al., 2006) and result from the interaction of misfolded SOD1 with mitochondrial partners (Israelson et al., 2010; Li et al., 2010 and references therein). Evidence is accumulating that MD takes place also as a consequence of the expression of other ALS-related proteins, some of which are directly linked to the function of these organelles. For instance, mutations in VCP account for about 2% of familial ALS and R155H/+ *VCP* knock-in mice show signs of motor damage due to muscle denervation and degeneration accompanied by extensive accumulation of abnormal mitochondria in the inter-myofibrillar space; in this model slow motor neuron loss is accompanied by TDP-43 accumulation and extensive aggregation of mitochondria (Yin et al., 2012; Nalbandian et al., 2013). In a 2013 study in VCP-deficient cells and in fibroblasts with *VCP* mutations, Bartolome et al. demonstrated that VCP deficiency causes significant mitochondrial uncoupling, leading to decreased mitochondrial membrane potential, increased mitochondrial oxygen consumption and reduction of cellular ATP production (Bartolome et al., 2013).

ALS may derive from mitochondrial DNA instability, as suggested in a study on a family with a late-onset phenotype including motor neuron disease and cognitive decline resembling frontotemporal dementia. Those patients carried a missense mutation in the CHCHD10 gene that encodes a mitochondrial protein located in the intermembrane space and enriched at cristae junctions. Muscle fibers from those patients are ragged-red and cytochrome c oxidase-negative with respiratory chain deficiency and abnormal assembly of complex V. Their fibroblasts have respiratory chain deficiency, mitochondrial ultrastructural alterations and fragmentation of the mitochondrial network (Bannwarth et al., 2014).

Neuronal mitochondrial abnormalities occur also in models of familial ALS associated with proteins that are (at least apparently) not related with these organelles. MD is observed together with oxidative damage and induction of mitophagy in the motor neuron-like NSC34 cell line expressing wild type or mutant TDP-43 (Duan et al., 2010; Hong et al., 2012). Mice expressing wild-type human TDP-43 show a pathology resembling ALS and motor neurons from these mice display cytoplasmic TDP-43 positive inclusions composed of mitochondria aggregates (Xu et al., 2010), that may arise from defective intracellular trafficking and result in the reduction of mitochondria at nerve terminals of neuromuscular junctions (Shan et al., 2010). However, in mice expressing the mutant TDP-43(A315T) protein, morphological abnormalities appear after the onset of transport defects (Magrané et al., 2014). In another study, MD in heterozygous TDP-43(A315TKi) animals is indicated by the formation of abnormal neuronal mitochondrial cristae and decrease in expression of Parkin and the fatty acid transporter CD36 along with an increase in fatty acids, HDL cholesterol, and glucose in the blood (Stribl et al., 2014). TDP-43 also perturbs ER-mitochondria interactions and this is associated with disruption in cellular Ca^2+^ homeostasis (Stoica et al., 2014). Disturbance of mitochondrial Ca^2+^ transport in ALS have been widely described in SOD1 models *in vitro* and *in vivo* by the group of B. Keller (Jaiswal and Keller, 2009; Jaiswal et al., 2009) and by others; this aspect has been recently reviewed in detail (Barrett et al., 2014; Tadic et al., 2014) and thus we will not discuss it in this paper.

Not much evidence of functional MD in FUS-linked ALS is reported. However, mitochondria are disorganized in post-mortem neurons from juvenile ALS patients (Huang et al., 2010) and shorter in cultured motor neurons expressing cytoplasmic FUS mutants (Tradewell et al., 2012). Furthermore, overexpression of ALS-linked human mutant FUS leads to Golgi fragmentation and mitochondria aggregation in rats (Huang et al., 2012).

How the overexpression or mutations of two proteins, TDP-43 and FUS, which are clearly involved in physiological mRNA metabolism, may result into MD in ALS is not clear at all.

One possibility is that misregulation of some step of RNA maturation leads to altered expression of proteins involved in mitochondria homeostasis. This may be the case for FUS, which transcriptionally regulates the expression of OS protection genes through the interaction with peroxisome proliferator-activated receptor coactivator 1-α (PGC-1α; Sánchez-Ramos et al., 2011), that is a known regulator of mitochondrial biogenesis and function. In this context, MD may arise indirectly from modulation of Sirtuins (class III histone deacetylases). In fact PGC-1α is downregulated in ALS patients and it is known that silencing of PGC1α reduces the expression of SIRT3, which is the main mitochondrial deacetylase with a number of substrates (including SOD2), whose main function is to counteract ROS production and detoxification (Bause and Haigis, 2013), while overexpression of SIRT3 stimulates the expression of PGC1α causing a further decrease of ROS in a positive feedback loop (Kong et al., 2010). Interestingly, both SIRT3 and PGC-1α protect against mitochondrial fragmentation and neuronal cell death in rat spinal cord motor neurons overexpressing ALS-linked mutant SOD1-G93A (Song et al., 2013). Furthermore, nuclear SIRT1 also induces the expression of OS response genes and promotes mitochondrial biogenesis by activating PGC-1α (Hall et al., 2013). Nonetheless, SIRT1 overexpression does not protect neurons against toxicity induced by mutant G93A-SOD1 (Valle et al., 2014 and reference therein).

Another possibility is that, similarly to mutant SOD1, TDP-43 and FUS have a pathological tendency to associate with mitochondria and to aggregate. Indeed, both wild type and a truncated form of TDP-43 were found localized in the mitochondria in NSC34 cells (Hong et al., 2012) and TDP-43 aggregates around mitochondria in a yeast model where altered mitochondrial respiratory activity is observed (Braun et al., 2011). Furthermore, FUS interacts with several proteins involved in mitochondrial metabolism and significantly reduces ATP levels in cells with FUS accumulation (Wang et al., 2015).

## Conclusions

**Figure 1:**
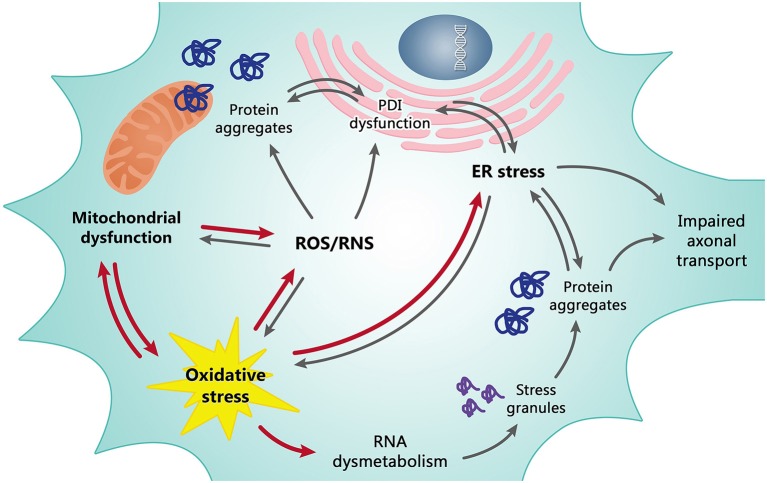
**Mitochondrial dysfunction and oxidative stress (OS) are tightly dependent on each other and are the basis of the redox dysregulation in ALS**. Increased production of ROS/RNS, the ER stress and, at least in part, transcriptional dysregulation and abnormal RNA processing are all consequences of mitochondrial dysfunction and OS contributing to death of motor neurons. In turn, these pathological events cause other correlated detrimental effects as the formation of misfolded protein leading to insoluble cytosolic and mitochondrial aggregates, impaired axonal transport and alteration of the enzymatic activity of PDI. The line between cause and effect of individual events is however often difficult to draw since they are all tightly dependent/connected. Red arrows may be considered as primary causes, grey arrows as secondary causes/effects.

Previous failures in translating antioxidant and mitochondria-protective strategies that were effective in the mutant SOD1 mouse model into positive clinical trials has cast a doubt on the real relevance of OS and MD in ALS and suggested that the SOD1 model represents only a fraction of a more heterogeneous population. However, new evidence has accumulated in recent years supporting the view that MD and OS play a role also in non-SOD1 ALS (Figure 1). This evidence is still somewhat sparse and often comes from studies in cell or invertebrate models that may represent only a few aspects of the human pathology. Nonetheless, while it has become clear that ALS is a complex disease with various presentation including an overlap with fronto-temporal dementia (Talbot, 2014), OS and MD may still represent a common denominator of motor neuron degeneration in ALS, and therefore a valuable target that encourages transfer of new knowledge from research into preclinical testing.

## Conflict of interest statement

The authors declare that the research was conducted in the absence of any commercial or financial relationships that could be construed as a potential conflict of interest.

## Abbreviations

ER: endoplasmic reticulum
ROS: reactive oxygen species
RNS: reactive nitrogen species
PDI: protein disulphide isomerase.
